# Supplementary material for: Effects of the Family Nurse Partnership on all eligible mothers: a data linkage cohort study in England
Source: PLoS One. 2025 Apr 3;20(4):e0320810. doi: 10.1371/journal.pone.0320810 (PMC11967931; doi:10.1371/journal.pone.0320810)
Supplement: S1 Table — (DOCX) [file pone.0320810.s001.docx]

## **S1 Table:** **Family Nurse Partnership site enrolment activity dates between April 2010 and March 2019 (mothers aged 13-19)**

| **FNP site name** | **Lower-tier Local Authority(ies)** | **Start date^$^** | **End date^$^** |
| --- | --- | --- | --- |
| North East |  |  |  |
| Durham & Darlington | Darlington, County Durham | 2009m8 | 2015m10 |
| Sunderland* | Sunderland | 2009m8 | 2019m3 |
| South Tyneside | South Tyneside | 2010m1 | 2018m1 |
| Middlesbrough | Middlesbrough, Redcar and Cleveland | 2012m3 2012m4 | 2015m11 2018m3 |
| Hartlepool | Hartlepool, Stockton-on-Tees | 2012m2 2012m4 | 2017m2 2017m12 |
| Northumberland | Northumberland | 2014m3 | 2016m9 |
| Newcastle upon Tyne | Newcastle-upon-Tyne | 2014m4 | 2016m9 |
| North Tyneside | North Tyneside | 2014m3 | 2016m3 |
| Gateshead | Gateshead | 2010m2 | 2019m3 |
| North West |  |  |  |
| Manchester* | Manchester | 2009m8 | 2016m9 |
| Blackpool | Blackpool | 2009m8 | 2019m3 |
| Cumbria* | Allerdale, Barrow-in-Furness, Carlisle, Copeland, Eden, South Lakeland | 2009m8 | 2016m8 |
| Liverpool* | Liverpool | 2009m8 | 2019m3 |
| Knowsley | Knowsley | 2009m8 | 2019m3 |
| Wirral | Wirral | 2009m8 | 2019m3 |
| Bolton | Bolton | 2011m11 | 2019m3 |
| Wigan | Wigan | 2011m12 | 2019m3 |
| Cheshire West | Cheshire West & Chester | 2012m2 | 2019m3 |
| Cheshire East | Cheshire East | 2012m9 | 2019m3 |
| Oldham | Oldham | 2014m8 | 2019m3 |
| Stockport | Stockport | 2014m8 | 2019m3 |
| Rochdale | Rochdale | 2014m8 | 2017m2 |
| Bury | Bury | 2014m12 | 2019m3 |
| Halton | Halton | 2014m7 | 2019m3 |
| Salford | Salford | 2014m12 | 2019m3 |
| St Helens | St Helens | 2014m12 | 2019m3 |
| Trafford | Trafford | 2015m1 | 2017m3 |
| Blackburn with Darwen | Blackburn with Darwen | 2015m5 | 2016m4 |
| Lancashire | Burnley, Preston | 2015m4 | 2017m4 |
| Sefton | Sefton | 2014m12 | 2016m9 |
| Tameside | Tameside | 2015m3 | 2019m3 |
| Warrington | Warrington | 2015m4 | 2019m3 |
| Yorkshire and Humber |  |  |  |
| Barnsley* | Barnsley | 2009m8 | 2015m11 |
| Calderdale | Calderdale | 2009m8 | 2016m2 |
| Hull* | Kingston upon Hull, city of | 2009m8 | 2017m6 |
| Leeds* | Leeds | 2009m8 | 2016m4 |
| Doncaster | Doncaster | 2009m8 | 2016m3 |
| Kirklees | Kirklees | 2009m8 | 2019m3 |
| Sheffield | Sheffield | 2009m8 | 2016m2 |
| Bradford & Airedale | Bradford | 2010m2 | 2019m3 |
| Rotherham | Rotherham | 2011m11 | 2016m12 |
| Wakefield | Wakefield | 2011m11 | 2019m3 |
| North & North East Lincolnshire | North East Lincolnshire, North Lincolnshire | 2012m3 | 2017m4 |
| East Riding | East Riding of Yorkshire | 2013m6 | 2019m3 |
| North Yorkshire | Scarborough | 2013m6 | 2014m12 |
| East Midlands |  |  |  |
| Derby City* | Derby | 2009m8 | 2019m3 |
| Nottingham City | Nottingham | 2009m8 | 2019m3 |
| Northamptonshire* | Corby, Daventry, East Northamptonshire, Kettering, Northampton, South Northamptonshire, Wellingborough | 2009m8 | 2019m3 |
| Derbyshire | Amber Valley, Bolsover, Chesterfield, Derbyshire Dales, Erewash, High Peak, North East Derbyshire, South Derbyshire | 2011m10 | 2019m3 |
| Leicester City | Leicester | 2011m9 | 2017m5 |
| Nottinghamshire | Ashfield, Bassetlaw, Broxtowe, Gedling, Mansfield, Newark and Sherwood, Rushcliffe | 2012m10 | 2019m3 |
| Lincolnshire | Boston, East Lindsey | 2014m8 | 2016m2 |
| West Midlands |  |  |  |
| Walsall* | Walsall | 2009m8 | 2016m9 |
| Stoke-on-Trent | Stoke-on-Trent, Newcastle-under-Lyme | 2009m8 | 2016m10 |
| Coventry* | Coventry | 2009m8 | 2019m3 |
| Birmingham*^1^ | Birmingham | 2013m1 | 2016m5 |
| Sandwell | Sandwell | 2009m8 | 2017m1 |
| Telford & Wrekin | Telford and Wrekin | 2009m8 | 2019m3 |
| Solihull | Birmingham, Solihull | 2009m8 | 2019m3 |
| Warwickshire North | North Warwickshire, Nuneaton and Bedworth | 2010m11 | 2019m3 |
| Dudley | Dudley | 2013m2 | 2019m3 |
| Staffordshire - Cannock and Tamworth | Cannock Chase, Tamworth | 2013m3 | 2016m8 |
| East Staffordshire | East Staffordshire | 2013m3 | 2017m4 |
| Shropshire | Shropshire | 2014m11 | 2019m3 |
| Wolverhampton | Wolverhampton | 2014m11 | 2017m7 |
| Warwickshire South & Rugby | Rugby, Stratford-on-Avon, Warwick | 2010m11 | 2019m3 |
| Worcestershire | Bromsgrove, Malvern Hills, Redditch, Worcester, Wychavon, Wyre Forest | 2015m9 | 2018m4 |
| East of England |  |  |  |
| South-East Essex* | Thurrock, Basildon, Castle Point, Rochford | 2009m8 2015m2 | 2016m10 2017m2 |
| Peterborough & Cambridgeshire | Peterborough, Cambridge, East Cambridgeshire, Fenland, Huntingdonshire, South Cambridgeshire | 2010m1 2011m12 | 2019m3 |
| Norfolk | Breckland, Broaland, Great Yarmouth, King's Lynn and West Norfolk, North Norfolk, Norwich, South Norfolk | 2010m1 | 2019m3 |
| Hertfordshire | Broxbourne, Dacorum, East Hertfordshire, Hertsmere, North Hertfordshire, St Albans, Stevenage, Three Rivers, Watford, Welwyn Hatfield | 2011m9 | 2016m2 |
| Suffolk | Ipswich, Suffolk Coastal, Waveney | 2010m10 2013m8 | 2018m3 2018m5 2019m3 |
| North Essex | Braintree, Colchester, Harlow, Tendring | 2014m11 | 2016m11 |
| Bedford & Bedfordshire | Bedford, Central Bedfordshire | 2015m1 | 2017m1 |
| Luton | Luton | 2015m9 | 2017m8 |
| Southend | Southend-on-Sea | 2009m8 | 2019m3 |
| London |  |  |  |
| Southwark* | Southwark | 2009m8 | 2019m3 |
| Tower Hamlets* | Tower Hamlets | 2009m8 | 2019m3 |
| Islington | Islington | 2009m8 | 2019m3 |
| Lambeth* | Lambeth | 2009m8 | 2019m3 |
| Ealing | Ealing | 2009m8 | 2019m3 |
| Waltham Forest & Redbridge | Redbridge, Waltham Forest | 2009m8 2013m10 | 2019m3 |
| West Central London | Hammersmith and Fulham, Kensington and Chelsea, Westminster | 2010m2 | 2019m3 |
| Lewisham | Lewisham | 2010m1 | 2019m3 |
| Barking & Dagenham | Barking & Dagenham | 2010m11 | 2015m11 |
| Croydon | Croydon | 2010m10 | 2019m3 |
| Haringey | Haringey | 2010m7 | 2019m3 |
| Barnet | Barnet | 2011m11 | 2019m3 |
| Hounslow | Hounslow | 2011m7 | 2019m3 |
| Enfield | Enfield | 2013m11 | 2019m3 |
| Newham | Newham | 2013m9 | 2019m3 |
| Sutton | Sutton | 2014m1 | 2017m6 |
| Brent | Brent | 2014m6 | 2016m7 |
| Greenwich | Greenwich | 2014m4 | 2019m3 |
| Hackney | Hackney, City of London | 2014m5 | 2019m3 |
| Bromley & Bexley | Bexley, Bromley | 2014m9 2014m10 | 2016m8 2019m3 |
| Camden | Camden | 2015m1 | 2018m7 |
| Wandsworth | Wandsworth | 2015m5 | 2019m3 |
| Merton | Merton | 2013m9 | 2019m3 |
| South East |  |  |  |
| Berkshire East* | Bracknell Forest, Slough, Windsor and Maidenhead | 2009m8 | 2016m2 |
| East Sussex - East | Hastings, Rother | 2009m8 | 2015m11 2015m12 |
| Milton Keynes | Milton Keynes | 2009m8 | 2016m10 |
| Southampton | Southampton | 2009m8 | 2019m3 |
| Medway | Medway | 2009m8 | 2015m12 |
| Oxfordshire | Cherwell, Oxford, South Oxfordshire, Vale of White Horse, West Oxfordshire | 2010m2 | 2019m3 |
| East Sussex - West | Eastbourne, Lewes, Wealden | 2010m11 2011m3 2011m12 | 2015m6 2015m11 2015m12 |
| West Sussex | Adur, Arun, Chichester, Crawley, Horsham, Mid Sussex, Worthing | 2011m9 | 2019m3 |
| Portsmouth | Portsmouth | 2011m11 | 2019m3 |
| Kent North | Gravesham, Swale, Thanet | 2012m2 2014m12 | 2017m3 2018m6 |
| Berkshire West | West Berkshire, Reading, Wokingham | 2012m11 | 2016m6 |
| Brighton & Hove | Brighton & Hove | 2012m10 | 2016m3 |
| Buckinghamshire | Aylesbury Vale, Chiltern, South Buckinghamshire, Wycombe | 2012m7 | 2019m3 |
| Kent South | Dover, Maidstone, Shepway, Tonbridge and Malling | 2013m2 2014m12 | 2017m10 2018m6 |
| Surrey | Elmbridge, Epsom and Ewell, Guildford, Mole Valley, Reigate and Banstead, Runnymede, Spelthorne, Surrey Heath, Tandridge, Waverley, Woking | 2014m11 | 2019m3 |
| Hampshire | Basingstoke and Deane, Fareham, Gosport, Hart, Havant, Rushmoor | 2015m3 2015m4 2015m8 2016m1 | 2017m4 2018m3 2018m8 2019m3 |
| South West |  |  |  |
| Plymouth | Plymouth | 2009m8 | 2019m3 |
| Cornwall & Isles of Scilly* | Cornwall, Isles of Scilly | 2009m8 | 2017m11 |
| Swindon | Swindon | 2009m8 | 2019m3 |
| Bath & North East Somerset | Bath & North East Somerset | 2013m2 | 2019m3 |
| Bristol & South Gloucestershire | Bristol, city of, South Gloucestershire | 2014m5 | 2019m3 |
| Bristol and North Somerset | North Somerset | 2014m8 | 2015m8 |
| Wiltshire | Wiltshire | 2014m11 | 2019m3 |

^$^Multiple start/end dates indicate different dates in different Local Authorities within FNP sites
