# Supplementary material for: Effects of the Family Nurse Partnership on all eligible mothers: a data linkage cohort study in England
Source: PLoS One. 2025 Apr 3;20(4):e0320810. doi: 10.1371/journal.pone.0320810 (PMC11967931; doi:10.1371/journal.pone.0320810)
Supplement: S2 Table — (DOCX) [file pone.0320810.s002.docx]

**S2 Table: ICD-10 code lists for maternal hospital admissions related to adversity, mental health, and chronic conditions**

Code lists were derived from the following studies:

- Harron K, Gilbert R, Fagg J, Guttmann A, van der Meulen J. Associations between pre-pregnancy psychosocial risk factors and infant outcomes: a population-based cohort study in England. *The Lancet Public Health* 2021; 6(2): e97-e105.
- Herbert A, Gilbert R, González-Izquierdo A, et al. Violence, self-harm and drug or alcohol misuse in adolescents admitted to hospitals in England for injury: a retrospective cohort study. BMJ Open. 2015;5(2):e006079.
- Hardelid P, Dattani N, Gilbert R. Estimating the prevalence of chronic conditions in children who die in England, Scotland and Wales: a data linkage cohort study. BMJ Open. 2014;4(8).
- Pearson RJ, Jay MA, Wijlaars LPMM, et al. Association between health indicators of maternal adversity and the rate of infant entry to Local Authority care in England: a longitudinal ecological study. *BMJ Open* 2020; 10(8): e036564.

Unplanned maternal hospital admissions with any of the ICD-10 codes in the following respective categories were considered to be adversity-related or mental health-related. Mothers with any hospital admissions relating to a chronic condition were considered to have a chronic condition.

| **Group** | **Description** | **ICD10 Code** |
| --- | --- | --- |
| **Adversity-related admissions** | |  |
| **Violence** | Maltreatment syndromes | T74 |
|  | Effects of other deprivation (extreme neglect) | T73 |
|  | Perpetrator of neglect and other maltreatment syndromes | Y06, Y07 |
|  | Assault by bodily force and sexual assault | Y04, Y05 |
|  | Other types of assault | X85-Y03, Y08-Y09 |
|  | Events of undetermined intent | Y20-Y34 |
|  | Examination and observation following other inflicted injury | Z04.5 |
|  | Examination and observation for other reasons: request for expert evidence | Z04.8 |
| **Self-harm** | Sequelae of intentional self-harm | Y87.0 |
|  | Intentional self-poisoning by and exposure to … drugs | X60-X63 |
|  | …other and unspecified drugs, medicaments and biological substances | X64 |
|  | …alcohol | X65 |
|  | …organic solvents and halogenated hydrocarbons and their vapours | X66 |
|  | …other gases and vapours | X67 |
|  | …pesticides | X68 |
|  | …other and unspecified chemicals and noxious substances | X69 |
|  | Intentional self-harm by… hanging, strangulation and suffocation | X70 |
|  | …drowning and submersion | X71 |
|  | …firearm discharge | X72-X74 |
|  | …explosive material | X75 |
|  | …smoke, fire and flames, or steam, hot vapours and hot objects | X76-X77 |
|  | …sharp/blunt objects | X78-X79 |
|  | ...jumping from a high place | X80 |
|  | …jumping or lying before a moving object, or crashing a motor vehicle | X81-82 |
|  | …other specified means | X83 |
|  | …unspecified means | X84 |
| **Substance misuse** | Mental and behavioural disorders due to psychoactive substance use | F11-F16, F19 |
|  | Finding of drugs not normally found in blood | R78.1-R78.5 |
|  | Poisoning by drugs, medicaments and biological substances | T36-T50 (not T50.6) |
|  | Poisoning, undetermined intent | Y10-Y14 |
|  | Drug rehabilitation | Z50.3 |
|  | Drug abuse counselling and surveillance | Z71.5 |
|  | Drug use | Z72.2 |
|  | Mental and behavioural disorders due to use of volatile solvents | F18 |
|  | Accidental poisoning by and exposure to noxious substances | X40–X44, X46-X49 |
|  | Poisoning by chemical or noxious substance, undetermined intent | Y16-Y19 |
|  | Special epileptic syndromes - (related to alcohol, drugs, etc.) | G40.5 |
|  | Blood-alcohol and blood-drug test | Z04.0 |
|  | Alcohol-induced pseudo-Cushing's syndrome | E24.4 |
|  | Mental and behavioural disorders due to use of alcohol | F10 |
|  | Degeneration of nervous system due to alcohol | G31.2 |
|  | Alcoholic polyneuropathy | G62.1 |
|  | Alcoholic myopathy | G72.1 |
|  | Alcoholic cardiomyopathy | I42.6 |
|  | Alcoholic gastritis | K29.2 |
|  | Alcoholic liver disease | K70 |
|  | Alcohol-induced acute pancreatitis | K85.2 |
|  | Alcohol-induced chronic pancreatitis | K86.0 |
|  | Maternal care for (suspected) damage to fetus from alcohol | O35.4 |
|  | Finding of alcohol in blood | R78.0 |
|  | Poisoning: antidotes and chelating agents, not elsewhere classified | T50.6 |
|  | Toxic effect of alcohol | T51 |
|  | Accidental poisoning by exposure to alcohol | X45 |
|  | Poisoning by exposure to alcohol, undetermined intent | Y15 |
|  | Evidence of alcohol involvement determined by blood alcohol level | Y90 |
|  | Evidence of alcohol involvement determined by level of intoxication | Y91 |
|  | Alcohol rehabilitation | Z50.2 |
|  | Alcohol abuse counselling and surveillance | Z71.4 |
|  | Alcohol use | Z72.1 |
| **Mental health conditions / behavioural disorders (excluding those falling under adversity or chronic conditions)** | |  |
|  | Organic, including symptomatic, mental disorders | F00-F09* |
|  | Schizophrenia, schizotypal and delusional disorders | F20-F29 |
|  | Mood [affective] disorders | F30-F39 |
|  | Neurotic, stress-related and somatoform disorders | F40-F48 |
|  | Behavioural syndromes associated with physiological disturbances and physical factors | F50-F59** |
|  | Disorders of adult personality and behaviour | F60-F69*** |
|  | Mental retardation | F70-F79 |
|  | Disorders of psychological development | F80-F89 |
|  | Behavioural and emotional disorders with onset usually occurring in childhood and adolescence | F90-F98 |
|  | Sedatives, hypnotics and antianxiety drugs | Y47 |
|  | Psychotropic drugs, not elsewhere classified | Y49 |
| **Chronic conditions (except those falling under mental health conditions)** | | |
| **Cancer/blood disorders** | Neoplasms | C00-C97, D00-D02, D05-D09, D12, D13, D14.1-D14.4, D15, D20, D32-D35, D37- D48, D63.0, E34.0, E88.3, G13.0, G13.1, G53.3, G55.0, G63.1, G73.1, G73.2, G94.1, M36.0, M36.1, M49.5, M82.0, M90.6, M90.7, N08.1, N16.1, Y43.1-Y43.3, Y84.2, Z08, Z51.0-Z51.2, Z54.1, Z54.2, Z85, Z86.0, Z92.3 |
|  | Immunological disorders | D80-D84, G53.2, Q98.0 |
|  | Anaemia and other blood disorders | D50, D56.0-D56.2, D56.4, D56.8, D56.9, D57.0-D57.2, D57.8, D58, D61.0, D61.9, D64, D66, D67, D68.0-D68.2, D68.4-D68.9, D69, D70-D76, M36.2-M36.4, M90.4, N08.2, Z86.2 |
| **Chronic infections** | HIV | B20-B24, F02.4, R75, Z21 |
|  | Tuberculosis | A15-A19, E35.0, K23.0, K67.3, K93.0, M01.1, M49.0, P37.0 |
|  | Other | A50, A81, B18, B37.1, B37.5, B37.6, B37.7, B38.1, B39.1, B40.1, B44.0, B44.7, B45, B46, B48.7, B50.0, B50.8, B51.0, B51.8, B52.8, B52.0, B55, B57.2-B57.5, B58.0, B59, B67, B69, B73, B74, B78.7, B90-B94, F02.1, K23.1, K93.1, M00, N33.0, P35.0- P35.2, P35.8, P35.9, P37.1 |
| **Respiratory** | Asthma and chronic lower respiratory disease | J41-J47 |
|  | Cystic fibrosis | E84, P75 |
|  | Injuries | S17, S27, S28, T27, T91.4 |
|  | Congenital anomalies | Q30-Q37, Q79.0 |
|  | Other | G47.3, J60-J70, J80-J86, J96.1, J98, P27, Y55.6, Z43.0, Z93.0, Z94.2 |
| **Metabolic/endocrine /digestive /renal /genitourinary** | Diabetes | E10-E14, G59.0, G63.2, I79.2, M14.2, N08.3, O24, Y42.3 |
|  | Other endocrine | E00, E03.0, E03.1, E07.1, E22.0, E23.0, E25, E26.8, E29.1, E31, E34.1, E34.2, E34.5, E34.8, G13.2, G73.5, Y42.1 |
|  | Digestive | K20, K21.0, K22, K23.8, K25-K28, K29.0, K29.1, K29.3-K29.9, K31, K50-K52, K55, K57, K59.2, K63.0-K63.3, K66, K72-K76, K80-K83, K85.0, K85.1, K85.8, K85.9, K86.1-K86.9, K87.0, K90, M07.4, M07.5, M09.1, M09.2, T86.4, Z43.2-Z43.4, Z46.5, Z90.3, Z90.4, Z93.2-Z93.5 |
|  | Renal/genitourinary | D63.8, G63.8, G99.8, I68.8, M90.8, N08.4, N00-N05, N07, N11-N15, N16.0, N16.2, N16.4, N16.5, N16.8, N18, N19, N20-N23, N25, N26, N28, N29, N31, N32, N33.8, N35, N36, N39.1, N39.3, N39.4, N40-N42, N70-N74, N80-N82, N85, N86, N87,N88, P96.0, T82.4, T83.1, T83.2, T83.4-T83.9, T85.5, T86.1, Y60.2, Y61.2, Y62.2, Y84.1, Z49, Z93.6, Z94.0, Z99.2 |
|  | Congenital anomalies of the digestive/renal/genitourinary system | Q38.0, Q38.3, Q38.4, Q38.6-Q38.8, Q39, Q40.2, Q40.3, Q40.8, Q40.9, Q41, Q42, Q43.1, Q43.3-Q43.7, Q43.9, Q44, Q45, Q50.0, Q51, Q52.0-Q52.2, Q52.4, Q54.0- Q54.3, Q54.8, Q54.9, Q55.0, Q55.5, Q56, Q60.1, Q60.2, Q60.4-Q60.6, Q61, Q62.0- Q62.6, Q62.8, Q63.0-Q63.2, Q63.8, Q63.9, Q64, Q79.2-Q79.5, Q87.8, Q89.1, Q89.2 |
|  | Injuries | S36, S37, S38, S39.6, S39.7, T06.5, T28, T91.5 |
|  | Other/unspecified | E66, G63.3, G99.0, M14.5, N92, Z86.3, Z93.8 |
| **Musculoskeletal/ skin** | Musculoskeletal/connective tissue | G55.1-G55.3, G63.5, G63.6, G73.7, J99.0, J99.1, L62.0, M05, M06, M07.0-M07.3, M07.6, M08, M09.8, M10-M13, M14.0, M14.6, M14.8, M30-M35, M40-M43, M45- M48,M50-M54, M60-M62, M63.8, M80.1-M80.9, M81.1-M81.9, M82.1, M82.8, M84.0-M84.2, M84.8, M84.9, M85, M86.3-M86.6, M89, M90.0, M91-M94, N08.5, Y45.4 |
|  | Skeletal injuries/amputations | S13, S22.0-S22.2, S22.5, S23, S32, S33, S68.3, S68.4, S68.8, S77, S78, S87, S88, S97, S98.0, S98.2-S98.4, T02, T04, T05, T20.3, T20.7, T21.3, T21.7, T22.3, T22.7, T23.2, T23.3, T23.6, T23.7, T24.3, T24.7, T25.2, T25.3, T25.6, T25.7, T29.3, T29.7, T30.3, T30.7, T31.2-T31.9, T32.2- T32.9, T87.3-T87.6, T91.2 T91.8, T92.6, T93.1, T93.4, T93.6, T94.0, T94.1, T95.0, T95.1, T95.4, T95.8, T95.9, Y83.5, Z89.1, Z89.2, Z89.5-Z89.8, Z97.1 |
|  | Chronic skin disorders | L10, L11.0, L11.8, L11.9, L12-L14, L28, L40-L45, L57, L58.1, L59, L87, L88, L90, 3 L92, L95, L93, L98.5, M09.0, Q80, Q81, Q87.0-Q87.5, Q89.4 |
|  | Congenital anomalies | Q18.8, Q65.0-Q65.2, Q65.8, Q65.9, Q67.5, Q68.2, Q68.3-Q68.5, Q71-Q73, Q74, Q75.3-Q75.9, Q76.1-Q76.4, Q77, Q78, Q79.6, Q79.8, Q82.0-Q82.4, Q82.9, Q86.2, Q89.7-Q89.9 |
| **Neurological** | Epilepsy | F80.3, G40.0-G40.4, G40.6-G40.9, G41, R56.8, Y46.0-Y46.6 |
|  | Cerebral palsy | G80-G83 |
|  | Injuries of brain, nerves, eyes or ears | S05-S08, S12, S14, S24, S34, S44, S54, S64, S74, S84, S94, T06.0- T06.2, T26, T90.4, T90.5, T91.1, T91.3, T92.4 |
|  | Chronic eye conditions | H05.1-H05.9, H13.3, H17, H18, H19.3, H19.8, H21, H26, H27, H28.0-H28.2, H31, H32.8, H33, H34, H35, H40, H42.0, H43, H44, H47, H54.0- H54.2, H54.4, T85.2, T85.3, Z44.2 |
|  | Chronic ear conditions | H60.2, H65.2-H65.4, H66.1-H66.3, H69.0, H70.1, H73.1, H74.0-H74.3, H75.0, H80, H81.0, H81.4, H83.0, H83.2, H90.0, H90.3, H90.5, H90.6, H91, Z45.3 |
|  | Perinatal conditions | P10, P21.0, P52, P57, P90, P91.1, P91.2, P91.6 |
|  | Congenital anomalies of neurological or sensory systems | Q00-Q07, Q10.4, Q10.7, Q11-Q12, Q13.0-Q13.4, Q13.8, Q13.9, Q14-Q16, Q75.0, Q75.1, Q85, Q86.0, Q86.1, Q86.8, Q90-Q93, Q95.2, Q95.3, Q97, Q99 |
|  | Other | F02.2, F02.3,G00-G09, G10-G12, G13.8, G14, G20-G23, G24.1-G24.9, G25-G30, G31.0-G31.1, G31.8, G31.9, G32-G37, G43-G46, G47.0-G47.2, G47.4-G47.9, G50- G52, G53.0, G53.1, G53.8, G54, G55.8, G56-G58, G59.8, G60, G61, G62.0, G62.2- G62.9, G64, G70, G71,G72.2-G72.9, G73.0, G73.3, G90-G93, G94.2, G94.8, G95, G96, G98, G99.1, G99.2, I60-I67, I68.0, I68.2, I69, I72.0, I72.5, T85.0, T85.1, Y46.7- Y46.8, Z98.2 |
| **Cardiovascular** | Congenital heart disease | Q20-Q26, Q89.3 |
|  | Other | I00-I28, I31-I39, I41, I42.0-I42.5, I42.7-I42.9, I43.0, I43.1, I43.2-I43.8, I44.1-I44.7, I45.1-I45.9, I46-I51, I52.8, I70-I71, I72.1-I72.4, I72.8, I72.9, I73-I77, I79.0, I79.1, I79.8, I81-I82, I98-I99, M03.6, N08.8, Q27, Q28, S26, T82.0-T82.3, T82.5-T82.9, T86.2, Y60.5, Y61.5, Y62.5, Y84.0, Z45.0, Z50.0, Z94.1, Z95 |
| **Codes indicating non-specific chronic conditions** | - | R62, R63.3, Z43.1, Z51.5, Z75.5, Z93.1, Z99.3 |

* Excluding F020-F024 (dementia codes)

** Excluding F51 (nonorganic sleep disorders) and F52 (sexual dysfunction, not caused by organic disorder or disease)

*** Excluding F64 (gender identity disorders (including transsexualism, transvestism, and “gender disorders”
