# Supplementary material for: Effects of the Family Nurse Partnership on all eligible mothers: a data linkage cohort study in England
Source: PLoS One. 2025 Apr 3;20(4):e0320810. doi: 10.1371/journal.pone.0320810 (PMC11967931; doi:10.1371/journal.pone.0320810)
Supplement: S3 Table — (DOCX) [file pone.0320810.s003.docx]

**S3 Table: ICD-10 code lists for child maltreatment and health care utilisation-related outcomes**

Code lists were derived from the following studies:

- Gilbert R, Fluke J, O’Donnell M, et al. Child maltreatment: variation in trends and policies in six developed countries. Lancet 2011
- Syed S, Ashwick R, Schlosser M, et al Predictive value of indicators for identifying child maltreatment and intimate partner violence in coded electronic health records: a systematic review and meta-analysis. Archives of Disease in Childhood 2021;106:44-53.
- Robling M, Lugg-Widger F, Cannings-John R, Sanders J, Angel L, Channon S, et al. The Family Nurse Partnership to reduce maltreatment and improve child health and development in young children: the BB:2–6 routine data-linkage follow-up to earlier RCT. Public Health Res 2021;9(2).

Unplanned hospital admissions with any of the ICD-10 codes in the following respective categories were considered to be maltreatment or injury related:

| **Group** | **Description** | **ICD-10 Code** | **Age restriction** |
| --- | --- | --- | --- |
| **Maltreatment and injury-related** | |  |  |
| **Injury & ingestion** | Injuries to the head (includes open wounds, fractures, crushing and dislocation) | S00-S09 | N/A |
|  | Injuries to the neck | S10-S19 | N/A |
|  | Injuries to the thorax | S20-S29 | N/A |
|  | Injuries to the abdomen, lower back, lumbar spine and pelvis | S30-S39 | N/A |
|  | Injuries to the shoulder and upper arm | S40-S49 | N/A |
|  | Injuries to the elbow and forearm | S50-S59 | N/A |
|  | Injuries to the wrist and hand | S60-S69 | N/A |
|  | Injuries to the hip and thigh | S70-S79 | N/A |
|  | Injuries to the knee and lower leg | S80-S89 | N/A |
|  | Injuries to the ankle and foot | S90-S99 | N/A |
|  | Injuries involving multiple body regions | T00-T07 | N/A |
|  | Injuries to unspecified part of trunk, limb or body region | T08-T14 | N/A |
|  | Effects of foreign body entering through natural orifice | T15-T19 | N/A |
|  | Burns and corrosions | T20-T32 | N/A |
|  | Frostbite | T33-T35 | N/A |
|  | Poisoning by drugs, medicaments and biological substances | T36-T50 | N/A |
|  | Toxic effects of substances chiefly non-medicinal as to source (sting, alcohol, solvents, etc.) | T51-T65 | N/A |
|  | Other and unspecified effects of external causes (effects of radiation, heat and light, hypothermia, electric shock, asphyxiation, food deprivation) | T66-T78 | N/A |
|  | Accidental poisoning by and exposure to noxious substances | X40-X49 | N/A |
| **Maltreatment** | Maltreatment syndromes | T74 | N/A |
|  | Neglect and abandonment | Y06 | N/A |
|  | Other maltreatment | Y07 | N/A |
|  | Effects of other deprivation (hunger, thirst, exhaustion due to exposure or excessive exertion) | T73 | N/A |
|  | Assault | X85-Y05 Y08-Y09 | N/A |
| **Maltreatment markers for infants <1yr** | Intracranial injuries | S06 | <1yr |
|  | Long-bone fractures | S42.2-S42.4, S42.7-S42.8, S52, S72, S82, T10, T12 | <1yr |
| **Maltreatment markers for children ≤4yrs** | Traumatic brain injuries | S06, S09.7-S09.8, T90.5 | ≤4yrs |
|  | Retinal haemorrhage | H35.6 | ≤4yrs |
|  | Rib fractures | T29-T32 | ≤4yrs |
