# Supplementary material for: Effects of the Family Nurse Partnership on all eligible mothers: a data linkage cohort study in England
Source: PLoS One. 2025 Apr 3;20(4):e0320810. doi: 10.1371/journal.pone.0320810 (PMC11967931; doi:10.1371/journal.pone.0320810)
Supplement: S4 Table — (DOCX) [file pone.0320810.s004.docx]

## **S4 Table: Maternal risk factors prior to 20 weeks of gestation used as covariates in adjusted models**

| **Maternal risk factor** | **Categorisation** |
| --- | --- |
| Date of delivery | Year / quarter-year |
| Maternal age at birth | 13-15, 16-17, 18-19, 20 years |
| Ethnicity | White, Black, South Asian, Mixed/Other, or Unknown |
| Area-level deprivation at birth | Quintile of the Index of Multiple Deprivation |
| Region of residence | South East, London, North West, East of England, West Midlands, South West, Yorkshire and the Humber, East Midlands, North East |
| Gestational age at booking | <10 weeks, 10-20 weeks, 20+ weeks |
| History of hospital attendances in the 2 years before 20 weeks of pregnancy: | Unplanned hospital admissions for adversity-related diagnoses*  Unplanned hospital admissions for mental health-related diagnoses*  Any hospital admission for chronic condition-related diagnoses*  Any A&E attendance  Repeated A&E attendance (4+ A&E attendances)  Did not attend ≥1 outpatient appointment |
| History of Social Care contacts before 20 weeks of pregnancy | Ever had a Child Protection Plan  Ever a Child Looked After |
| Educational risk factors before 20 weeks of pregnancy | Ever recorded as having Special Educational Needs provision  Ever recorded as having Free School Meals (eligible, applies for and receives)  Ever in the most deprived IDACI decile  Ever excluded from school, in a pupil referral unit, or alternative provision  Ever persistently absent (≥10% of possible sessions)  Achieved expected levels at Key Stage 2 Mathematics / English ^$^  Achieved 5 A*-Cs at GSCE level^^^ |

* see Appendix Tables S2-S3; ^$^ At age 11 years. Only available until 2013/14. ^^^ Amongst those who were aged ≥16 at the start of the academic year in which they reached 20 weeks of pregnancy; IDACI: Income Deprivation Affecting Children Index
