# Supplementary material for: Effects of the Family Nurse Partnership on all eligible mothers: a data linkage cohort study in England
Source: PLoS One. 2025 Apr 3;20(4):e0320810. doi: 10.1371/journal.pone.0320810 (PMC11967931; doi:10.1371/journal.pone.0320810)
Supplement: Attachment — Submitted filename: comment for plos one.docx [file pone.0320810.s006.docx]

1. Very good research question is raised
2. Regarding Healthy pregnancies, improving child development, and fulfilling their own aspirations and ambitions might have no difference in urban and educated community, but I believe it will have difference in rural and illiterate community like undeveloped countries.
3. Suggest this study to be conducted in rural community

**Data sources and linkage (page-3)**

1. I agree with the data source you mentioned, but I didn’t seen the link between the data source and the subject under study (family nurse partnership)?

…….We obtained information about FNP participation dates from the FNP Information System (98.5% of FNP mothers were linked to a HES record). What was the data? How was this data liked to FNP? What tasks were done while in the partnership to compare with the counterpart (who did not take part in FNP).

I believe it would be soundly if the source of the data and the research question has direct link. Did you have what you want from the data source? I believe you know it have much information than me. I raised this question by the hesitating on the appropriateness of the data source for the conclusion and the finding.
