# Supplementary material for: Effects of the Family Nurse Partnership on all eligible mothers: a data linkage cohort study in England
Source: PLoS One. 2025 Apr 3;20(4):e0320810. doi: 10.1371/journal.pone.0320810 (PMC11967931; doi:10.1371/journal.pone.0320810)
Supplement: Attachment — Submitted filename: PLOS One review of Englsih data linkage study.docx [file pone.0320810.s007.docx]

Thank you for the opportunity to review this manuscript. While it addresses interesting issues, it fails to include fundamental information for readers to have in interpreting the results of this study:

1. FNP is a licensed program in which those licensed to deliver it are committed to protecting the licensed material. Thus, spill-over effects are not likely to be discerned, although local areas may choose to visit families in need more frequently than through usual health visiting and midwifery, and to use the general approach employed by FNP nurses during later periods of care, when FNP was not delivered.
2. It would be useful to examine the frequency of health-visiting and midwifery contacts before, during, and after FNP was delivered in specific areas. That would be useful data to use in this report, if the data are available. Evidence from the BB0-2 report indicated that health-visitors paid many more visits (~16) to the control group during the trial-period^1^ than the maximum typically delivered to families by health visitors prior to the trial (a maximum of 3). It’s not clear how many visits were paid to families with greater needs. Are health visitors and midwives currently visiting families in need today than before FNP went into effect?
3. The reader needs to see the degree to which those cases used in the final analyses were comparable on “baseline” characteristics used for comparing FNP enrollees to those adolescent mothers not enrolled, like the authors of this paper reported in a BMJ report published earlier this year. For a study like this one, these critical data points are needed by reviewers in the main body of the report to discern clearly which groups are being compared and the degree to which they were comparable.
4. The authors should acknowledge explicitly that FNP today focuses on those with heightened needs, given that the Cardiff trial results led to efforts on the part of FNP leaders to target the program on those with greater needs. Please search for the Adapt report.
5. Greater specification of the differences between those who received FNP and the rest of the population < 20Y will be important to report toward the beginning of this manuscript.
6. Note that a German trial of this program focused on those with overlapping needs but combined two versions of the program in the original reports: one delivered by midwives throughout the entire program period (pregnancy through child-age 2 – the Continuous model, and a second in which it was delivered by midwives from pregnancy through 6-months postpartum and then by social workers through child-age 2 - the tandem model. Early reports that combined these two models produced equivocal results.^2^ Evidence is now accumulating that the version of the program delivered by midwives continuously is beginning to show that the midwife-only model is superior to the tandem model. ^3^ The point is that the German replicators focused on those with overlapping needs predictive of child maltreatment. The same can be said about trials of this program conducted in the Netherlands^4-6^ and British Columbia,^7^ which again focused on those with overlapping needs, not simply maternal age <20. Note that the Netherlands and German trials were conducted in countries with extensive health and social care systems – like the UK.
7. It’s thus not surprising that FNP showed no evidence of beneficial carry-over effects, given the absence of detailed data on those served by FNP and the lack of comparability of those included in the comparison group. While the authors have made statistical adjustments using data available through data-linkage, the sources of such data are crude and do not account for unmeasured differences.
8. Moreover, questions should be raised about the choice of outcomes in this study: a) unplanned hospitalizations through child-age two, b) differences in child admissions for maltreatment/injury-related diagnoses and c) maternal admissions for adversity-related diagnoses. FNP nurses encourage parents to take their children to A&E to rule out internal injuries following an accident, and for mothers to do the same for themselves -- which will increase these types of admissions on the part of FNP-visited families. A better measure of child functioning (related to child maltreatment) is the *number of days* children were hospitalized with injuries, which was found to distinguish treatment and control groups in the US Memphis^8^ and the Canadian BC trials,^7^ which reflects injury severity, and which is less susceptible to FNP-visited families using GP and A&E care to protect their children’s health.
9. These issues should be addressed in the introduction of the manuscript and the discussion.
10. Moreover, the introduction of the manuscript should include the school-readiness finding from the BB2-6 report as well as the maternally reported language effects from BB0-2.
11. The rest of the manuscript depends upon the starting point – that is addressing the differences between those enrolled in the program and the rest of the population <20Y in comparing FNP enrollees to the rest of the populations within areas that delivered FNP.
12. Finally, the manuscript needs a careful editorial review for spelling and grammar.

References

1. Robling, M., Bekkers, M. J., Bell, K., Butler, C. C., Cannings-John, R., Channon, S., ... & Torgerson, D.	(2016). Effectiveness of a nurse-led intensive home-visitation programme for first-time teenage	mothers (Building Blocks): a pragmatic randomised controlled trial. The Lancet, 387(10014), 146-	155. 10.1016/S0140-6736(15)00392-X
2. Kliem, S., & Sandner, M. (2021). Prenatal and infancy home visiting in Germany: 7-year outcomes of a	randomized trial. Pediatrics, 148(2). <https://doi.org/10.1542/peds.2020-049610>
3. Conti, G., Kliem, S., & Sandner, M. (2024). Early Home Visiting Delivery Model and Maternal and Child	Mental Health at Primary School Age. AEA Papers and Proceedings, 114, 401–406.
4. Mejdoubi, J., van den Heijkant, S. C., van Leerdam, F. J., Heymans, M. W., Hirasing, R. A., & Crijnen, A. A. (2013). Effect of nurse home visits vs. usual care on reducing intimate partner violence in young high-risk pregnant women: a randomized controlled trial. PloS one, 8(10), e78185. https://doi.org/10.1371/journal.pone.0078185
5. Mejdoubi, J., van den Heijkant, S. C., van Leerdam, F. J., Crone, M., Crijnen, A., & HiraSing, R. A. (2014). Effects of nurse home visitation on cigarette smoking, pregnancy outcomes and breastfeeding: a randomized controlled trial. Midwifery, 30(6), 688-695.	https://doi.org/10.1016/j.midw.2013.08.006
6. Mejdoubi, J., van den Heijkant, S. C., van Leerdam, F. J., Heymans, M. W., Crijnen, A., & Hirasing, R. A.	(2015). The effect of VoorZorg, the Dutch nurse-family partnership, on child maltreatment and development: a randomized controlled trial. PLoS One, 10(4), e0120182. https://doi.org/10.1371/journal.pone.0120182
7. Catherine, N. L., MacMillan, H., Cullen, A., Zheng, Y., Xie, H., Boyle, M., ... & Waddell, C. (2024).	Effectiveness of nurse‐home visiting in improving child and maternal outcomes prenatally to age two years: a randomised controlled trial (British Columbia Healthy Connections Project). Journal of Child Psychology and Psychiatry, 65(5), 644-655. <https://doi.org/10.1111/jcpp.13846>
8. Kitzman, H., Olds, D. L., Henderson, C. R., Hanks, C., Cole, R., Tatelbaum, R., ... & Barnard, K. (1997). Effect of prenatal and infancy home visitation by nurses on pregnancy outcomes, childhood injuries, and repeated childbearing: a randomized controlled trial. JAMA, 278(8), 644-652. doi:10.1001/JAMA.1997.03550080054039
