# Supplementary material for: Effects of the Family Nurse Partnership on all eligible mothers: a data linkage cohort study in England
Source: PLoS One. 2025 Apr 3;20(4):e0320810. doi: 10.1371/journal.pone.0320810 (PMC11967931; doi:10.1371/journal.pone.0320810)
Supplement: Attachment — Submitted filename: Response to reviewers.docx [file pone.0320810.s008.docx]

Dear Editors,

Thank you for the time you and the reviewers have taken to consider our paper. We have provided point by point responses to the comments, within the table below.

I hope that these responses will satisfy the queries that have been raised, but I would be happy to further address any additional issues that are raised.

Many thanks,
Katie

| **Editor/reviewer comment** | **Author response** |
| --- | --- |
| 1. Please ensure that your manuscript meets PLOS ONE's style requirements, including those for file naming. | This has been done. |
| 2. Please note that PLOS ONE has specific guidelines on code sharing for submissions in which author-generated code underpins the findings in the manuscript. In these cases, we expect all author-generated code to be made available without restrictions upon publication of the work. Please review our guidelines at <https://journals.plos.org/plosone/s/materials-and-software-sharing#loc-sharing-code> and ensure that your code is shared in a way that follows best practice and facilitates reproducibility and reuse. | Code will be made available via our GitHub site: We have now linked to this in the data availability statement. |
| 3. Please state what role the funders took in the study. | ""The funders had no role in study design, data collection and analysis, decision to publish, or preparation of the manuscript."" |
| 4. We note that you have provided funding information that is not currently declared in your Funding Statement. However, funding information should not appear in the Acknowledgments section or other areas of your manuscript. We will only publish funding information present in the Funding Statement section of the online submission form.  Please remove any funding-related text from the manuscript and let us know how you would like to update your Funding Statement. Please include your amended statements within your cover letter; we will change the online submission form on your behalf. | The funding statement should be as follows: “This study was supported by funding from NIHR Health Services Delivery & Research (17/99/19), and the authors were in part supported by the NIHR through the Great Ormond Street Hospital Biomedical Research Centre and the NIHR Children and Families Policy Research Unit and Senior Investigator award for RG.”  We have edited the Acknowledgements section to remove funding. |
| 5. In the online submission form, you indicated that we are unable to share the individual data used for this study. HES and FNP data can be requested through NHS Digital and NPD can be requested through the Department for Education.  All PLOS journals now require all data underlying the findings described in their manuscript to be freely available to other researchers, either 1. In a public repository, 2. Within the manuscript itself, or 3. Uploaded as supplementary information. This policy applies to all data except where public deposition would breach compliance with the protocol approved by your research ethics board. If your data cannot be made publicly available for ethical or legal reasons (e.g., public availability would compromise patient privacy), please explain your reasons on resubmission and your exemption request will be escalated for approval. | Individual level data cannot be made available under our ethics approval, or data sharing agreements with NHS England and the Department for education, and these data can only be assessed on secure servers, with specific project and researcher permissions. Public availability would compromise patient privacy. |
| 6. Please include captions for your Supporting Information files at the end of your manuscript, and update any in-text citations to match accordingly. | This has now been completed. |
| Reviewer #1: the manuscript is technically good, presented good introduction, method and data source. the result interpretation is soundly. the conclusion is soundly and as per the result. but the nature of the study might need further testing in different populations. for instance, rural communities. | Thank you for these comments. We agree that the analysis could be repeated in specific populations and have now included this as a suggest for further research in the discussion. |
| Reviewer #2: Thank you for the opportunity to review this manuscript. While it addresses interesting issues, it fails to include fundamental information for readers to have in interpreting the results of this study:  1. FNP is a licensed program in which those licensed to deliver it are committed to protecting the licensed material. Thus, spill-over effects are not likely to be discerned, although local areas may choose to visit families in need more frequently than through usual health visiting and midwifery, and to use the general approach employed by FNP nurses during later periods of care, when FNP was not delivered. | We agree with the point, which is that practitioners outside the FNP may have changed practice (e.g. though visiting families in need more frequently) whilst FNP was in place, or through extending usual local services after the FNP was finished. We have tried to capture these points in Table 1, and have changed the wording in the introduction to make clear that the FNP is a licensed program.  The text now states:  “The FNP programme is licensed, and involves protection of licensed materials. Qualitative research suggests that professionals are concerned about the licensed nature of FNP and not being able to share freely with wider health visiting colleagues or to use it to change practice across services.[16] Despite this, there may still be benefits to other services, for example if trained family nurses take on other roles and disseminate trauma-informed approaches, either while the FNP is being delivered, or after it has stopped being delivered in a local area.” |
| 2. It would be useful to examine the frequency of health-visiting and midwifery contacts before, during, and after FNP was delivered in specific areas. That would be useful data to use in this report, if the data are available. Evidence from the BB0-2 report indicated that health-visitors paid many more visits (~16) to the control group during the trial-period1 than the maximum typically delivered to families by health visitors prior to the trial (a maximum of 3). It’s not clear how many visits were paid to families with greater needs. Are health visitors and midwives currently visiting families in need today than before FNP went into effect? | We agree this would be very insightful. Unfortunately, national data on health visiting only began to be collated in 2016, and is very patchy. National data on midwifery contacts are not available.  Your point stands – ideally we would like to know if health visitors/midwives are visiting families more often now than before FNP was commissioned. Again though this is very difficult to measure, firstly from the data perspective, but also taking into account cuts to services and workforce which have affected how health visiting services are delivered over time.  The data from the BB0-2 report does support our hypothesis that there might be spillover effects from the FNP, and we have now included this in the introduction. |
| 3. The reader needs to see the degree to which those cases used in the final analyses were comparable on “baseline” characteristics used for comparing FNP enrollees to those adolescent mothers not enrolled, like the authors of this paper reported in a BMJ report published earlier this year. For a study like this one, these critical data points are needed by reviewers in the main body of the report to discern clearly which groups are being compared and the degree to which they were comparable. | Thank you for this point. We agree this is important – this comparison of characteristics is given in Table 2. We have not included FNP mothers as a separate group in this table, as we are not analysing this group separately, only as within the “Mothers during enrolment period” group. We know that FNP mothers will be more vulnerable, and this has been shown in the BMJ PH paper you mention. We have now made this point in the introduction, and referred to this paper.  We also discuss in detail within the introduction the fact that the current study aims to overcome problems associated with selectivity in the FNP enrolled mothers, by taking the approach of evaluating the effect on all eligible mothers. |
| 4. The authors should acknowledge explicitly that FNP today focuses on those with heightened needs, given that the Cardiff trial results led to efforts on the part of FNP leaders to target the program on those with greater needs. Please search for the Adapt report. | We have now acknowledged this specifically in the introduction, and referenced the ADAPT evaluation. |
| 5. Greater specification of the differences between those who received FNP and the rest of the population < 20Y will be important to report toward the beginning of this manuscript. | We have now described the characteristics of those who receive the FNP and the rest of the eligible population in the introduction. We could explicitly report the differences within the study population, but feel that this is missing the point – we are interested in the overall effect of the program on all eligible, not on the differences between those enrolled and the non-enrolled eligible (which has been reported separately). |
| 6. Note that a German trial of this program focused on those with overlapping needs but combined two versions of the program in the original reports: one delivered by midwives throughout the entire program period (pregnancy through child-age 2 – the Continuous model, and a second in which it was delivered by midwives from pregnancy through 6-months postpartum and then by social workers through child-age 2 - the tandem model. Early reports that combined these two models produced equivocal results.2 Evidence is now accumulating that the version of the program delivered by midwives continuously is beginning to show that the midwife-only model is superior to the tandem model. 3 The point is that the German replicators focused on those with overlapping needs predictive of child maltreatment. The same can be said about trials of this program conducted in the Netherlands4-6 and British Columbia,7 which again focused on those with overlapping needs, not simply maternal age <20. Note that the Netherlands and German trials were conducted in countries with extensive health and social care systems – like the UK. | Thank you for these references to other studies of the FNP. We not gone into a lengthy discussion of the effectiveness of different versions of the FNP on the enrolled population, since this is not the emphasis of this paper. These discussions have been given more detail elsewhere, see e.g. <https://www.journalslibrary.nihr.ac.uk/hsdr/BVDW6447> |
| 7. It’s thus not surprising that FNP showed no evidence of beneficial carry-over effects, given the absence of detailed data on those served by FNP and the lack of comparability of those included in the comparison group. While the authors have made statistical adjustments using data available through data-linkage, the sources of such data are crude and do not account for unmeasured differences. | We agree that it might not be surprising that there was no evidence of beneficial carry-over effects, but feel that it is still important to publish negative results, to add to the body of current evidence (which currently does not include assessments of the total effect of FNP on all eligible mothers).  We do provide as much detail as possible on the comparison groups in this study, which are those who were eligible before, during and after FNP implementation (note – we are not comparing outcomes specifically for those enrolled, which is the group that you refer to). These details are given in Table 2, and include information on age, ethnicity, deprivation, history of hospital attendances (including for mental health), and educational and social care history). As the reviewer notes, there were some differences. For example, more mothers during the enrolment period were living in the most deprived areas than those eligible mothers prior to enrolment. However, these characteristics were adjusted for within analyses. Whilst we agree that these characteristics are crude, we have discussed the implications of this in the discussion, and note that we cannot rule out unmeasured confounding. |
| 10. Moreover, the introduction of the manuscript should include the school-readiness finding from the BB2-6 report as well as the maternally reported language effects from BB0-2. | Thank you for this suggestion. We have now included these results in the introduction. |
| 11. The rest of the manuscript depends upon the starting point – that is addressing the differences between those enrolled in the program and the rest of the population <20Y in comparing FNP enrollees to the rest of the populations within areas that delivered FNP. | The difference between those enrolled in the FNP and those eligible to be enrolled has been reported previously and is not the focus of this paper. We know that there are differences – and this is what makes evaluating the effectiveness of the programme challenging, as we cannot adjust for all of these differences in observational data sources.  To overcome this problem, we have taken a different approach – which is to ask, what is the overall effect of the FNP on the population of eligible mothers, irrespective of who is actually enrolled? This answers a different question – but still an important one – which is whether commissioning the FNP in a local area but only offering it to 1 in 4 eligible mothers has an overall positive or negative effect on all those who could stand to benefit from increased levels of support. |
| 12. Finally, the manuscript needs a careful editorial review for spelling and grammar. | We have done this. |
