# Supplementary material for: Effects of the Family Nurse Partnership on all eligible mothers: a data linkage cohort study in England
Source: PLoS One. 2025 Apr 3;20(4):e0320810. doi: 10.1371/journal.pone.0320810 (PMC11967931; doi:10.1371/journal.pone.0320810)
Supplement: Attachment — Submitted filename: Response to reviewers R2.docx [file pone.0320810.s009.docx]

Dear Editors

Thank you for the additional reviews on our manuscript. The final reviewer had two main points, which we have responded to below.

*Comment 1: Overall, the article presents its methodology and study limitations clearly. This study reports rather unoptimistic findings: there is no evidence of differences in unplanned hospital admissions between children born during the Family Nurse Partnership (FNP) period, those born after FNP was implemented, and those born before FNP started. Although I initially found it unclear why unplanned hospital admissions were chosen as the primary outcome, the authors explained in the methods section that this decision was based on findings from a previous study, which makes sense.*

Response: Thank you for this positive feedback.

*To be honest, I am impressed by the authors' courage in presenting such disappointing data regarding the FNP program. Therefore, I believe the discussion section could be more in-depth or inspiring, which might enhance the readability of the article. For example, the second paragraph of the introduction mentions that the FNP program implemented in the United States was effective, making one curious about the differences in implementation between the UK and the US that might have led to the variation in outcomes. Additionally, I am curious about incorporating cost-effectiveness data, which might help readers assess the feasibility of the FNP program.*

Response: We agree that we could give more information in the discussion section about why there are differences in the evidence on the effectiveness of the FNP in the US and the UK, and have now amended the text. Although looking at cost-effectiveness is beyond the scope of this study, we also now refer to a cost-effectiveness study of the FNP in England, for context.

We hope that these changes satisfy the reviewer and the editors, but welcome any further feedback.

Kind regards,

Katie Harron
